# Comparative analysis of root transcriptome profiles of two pairs of drought-tolerant and susceptible rice near-isogenic lines under different drought stress

**DOI:** 10.1186/1471-2229-11-174

**Published:** 2011-12-02

**Authors:** Ali Moumeni, Kouji Satoh, Hiroaki Kondoh, Takayuki Asano, Aeni Hosaka, Ramiah Venuprasad, Rachid Serraj, Arvind Kumar, Hei Leung, Shoshi Kikuchi

**Affiliations:** 1 Plant Genome Research Unit, Agrogenomics Research Center, National Institute of Agrobiological Sciences (NIAS), Kan'non dai 2-1-2, Tsukuba, Ibaraki, 305-8602, Japan; 2 Rice Research Institute of Iran in Mazandaran, POBox 145, Postal-Code 46191-91951, Km8 Babol Rd., Amol, Mazandaran, Iran; 3 International Rice Research Institute, DAPO Box 7777, Metro Manila 1301, Philippines; 4 Africa Rice Centre (AfricaRice), Ibadan station, c/o IITA, PmB 5320 Oyo road, Nigeria; 5 International Centre for Agricultural Research in the Dry Areas (ICARDA), POBox 5466, Aleppo, Syria

## Abstract

**Background:**

Plant roots are important organs to uptake soil water and nutrients, perceiving and transducing of soil water deficit signals to shoot. The current knowledge of drought stress transcriptomes in rice are mostly relying on comparative studies of diverse genetic background under drought. A more reliable approach is to use near-isogenic lines (NILs) with a common genetic background but contrasting levels of resistance to drought stress under initial exposure to water deficit. Here, we examined two pairs of NILs in IR64 background with contrasting drought tolerance. We obtained gene expression profile in roots of rice NILs under different levels of drought stress help to identify genes and mechanisms involved in drought stress.

**Results:**

Global gene expression analysis showed that about 55% of genes differentially expressed in roots of rice in response to drought stress treatments. The number of differentially expressed genes (DEGs) increased in NILs as the level of water deficits, increased from mild to severe condition, suggesting that more genes were affected by increasing drought stress. Gene onthology (GO) test and biological pathway analysis indicated that activated genes in the drought tolerant NILs IR77298-14-1-2-B-10 and IR77298-5-6-B-18 were mostly involved in secondary metabolism, amino acid metabolism, response to stimulus, defence response, transcription and signal transduction, and down-regulated genes were involved in photosynthesis and cell wall growth. We also observed gibberellic acid (GA) and auxin crosstalk modulating lateral root formation in the tolerant NILs.

**Conclusions:**

Transcriptome analysis on two pairs of NILs with a common genetic background (~97%) showed distinctive differences in gene expression profiles and could be effective to unravel genes involved in drought tolerance. In comparison with the moderately tolerant NIL IR77298-5-6-B-18 and other susceptible NILs, the tolerant NIL IR77298-14-1-2-B-10 showed a greater number of DEGs for cell growth, hormone biosynthesis, cellular transports, amino acid metabolism, signalling, transcription factors and carbohydrate metabolism in response to drought stress treatments. Thus, different mechanisms are achieving tolerance in the two tolerant lines.

## Background

Water scarcity is one of the most pressing issues facing agriculture today. In many countries, water for agriculture consumes about 70% of the total fresh water use. To meet the needs of a growing population, more food must be produced with less water [1]. Rice (*Oryza sativa *L.) is the primary source of food for more than half of the world's population. Rice is cultivated in highly diverse situations that range from flooded wetland to rainfed dryland [2]. Irrigated rice which accounts for 55 percent of the world rice area provides 75% of global rice production and consumes about 90% of the freshwater resources used for agriculture in Asia [3]. Water deficit is therefore a key constraint that affects rice production in different countries. Severe drought can reduce seriously rice production, leading to catastrophic crop failure [4]. There is a need to improve drought tolerance in rice to have sustainable rice production in water-limiting areas [5]. An understanding of the underlying physiological and molecular mechanisms is necessary to improve the adaptation of rice varieties to drought-prone environments [5,6]. Progress has been made in detecting large effect quantitative trait loci (QTL) conferring drought tolerance in lowland and irrigated rice [5]. Still relatively limited information is available about the genetics and molecular control of drought tolerance.

Previous studies on genetics of drought tolerance in rice were primarily based on the analysis of mapping populations derived from parents of contrasting level of drought tolerance [7-9]. However, the heterogeneous genetic backgrounds of tolerant and susceptible germplasm often obscure the relationship between genetic variation and drought tolerance phenotypes. A more desirable approach is to use genetic stocks with a common genetic background but contrasting levels of tolerance to drought stress. Through selection in IRRI's drought breeding program, a set of advanced backcross lines was developed by backcrossing Aday Selection (AdaySel), a traditional variety to popular variety IR64 [10]. IR64 is the most widely grown rice variety in the tropical areas; it carries many valuable agronomic traits but is highly sensitive to drought stress [11]. Two pairs of NILs in the background of IR64 with contrasting drought tolerance were selected from [12]: a) IR77298-14-1-2-B family: IR77298-14-1-2-B-10 (highly drought-tolerant) vs IR77298-14-1-2-B-13 (susceptible), and b) IR77298-5-6-B family: IR77298-5-6-B-18 (moderately drought-tolerant) and IR77298-5-6-B-11 (highly susceptible). These advanced backcross lines are considered pre-near isogenic lines because they are sister lines derived from a single family segregating for drought tolerance.

One important aspect for understanding drought tolerance is the response of root growth and development to water-deficit conditions [13]. Roots are important for maintaining crop yields, vital when plants are grown in soils containing insufficient supplies of water or nutrients [14], and one of the primary sites for stress signal perception that initiates a cascade of gene expression responses to drought [15,16]. Previous studies showed that plant growth largely depends on the severity of the stress; mild water deficit leads to growth inhibition of leaves and stems, whereas roots may continue to elongate [17]. Furthermore, root architecture is a key trait for dissecting the genotypic differences in rice responses to water deficit [13]. A variety of studies were carried out on the gene expression patterns of roots in common bean [18], sunflower [19], *Arabidopsis *[20,21], maize [22] and other plants under drought stress. Gene expression profiles of upland and lowland rice for drought stress have been reported [23,24], but these studies focused on comparing gene expression profiles of genotypes at seedling stage in a single stress condition. Currently, little is known about expression patterns in root under different levels of water deficit in drought-tolerant and susceptible genotypes at reproductive stage. In this study, we used the Agilent 4 × 44 K oligoarray system to conduct transcript profiling in root of two pairs of rice NILs exhibiting large differences in their yield and physiological and phenological traits under drought stress at reproductive stage. Our results suggest a greater number of DEGs in roots of highly tolerant NIL, IR77298-14-1-2-B-10 compared to other NILs in response to severe drought stress. Genes related to cell growth were mostly down-regulated, while those related to ABA biosynthesis, proline metabolism, ROS-scavenging enzymes and carbohydrate metabolism were highly activated in tolerant NILs. Despite their common genetic background (~97%) as backcross progeny from Aday Sel × IR64, the two pairs of NILs show distinctive differences in their gene expression profiles in response to drought stress.

## Results and discussion

### Experimental design and root traits analysis

In this study, drought stress was imposed by initiating soil dry down protocol starting 35 days after seeding (DAS) and dried down until the pot reaches targeted fraction of transpirable soil water (FTSW) [25]. Several studies have shown that FTSW can be linked to variables describing plant water status such as midday leaf water potential, leaf relative water content and stomatal conductance [25,26]. Water regimes were 0.2 FTSW (severe stress), 0.5 FTSW (mild stress) and 1.0 FTSW (as control). Data on root characteristics such as number of roots per plant, root volume, roots dry weight, maximum root length and root thickness were recorded. Both the stress and control treatments had four replications each arranged as randomized complete block design (RCBD). Compared to the well-watered control condition, the severe stress treatment showed a large reduction in the number of roots per plant (54%), root volume (65%), and root dry weight (61%); while there was a significant increase in maximum root length (64%) and a slight increase in root thickness (3%) under stress relative to the non-stress (Table 1). The tolerant NIL in the IR77298-14-1-2-B family had a significantly higher number of roots, greater root thickness, and greater root dry weight than the susceptible NIL under stress but not under non-stress. In the IR77298-5-6-B family the tolerant NIL exhibited significantly higher rooting depth than the susceptible NIL under non-stress conditions only. Accordingly, the tolerant NILs in both families generally showed higher rooting depth, number of roots, root volume, and root dry weight than the corresponding susceptible NILs. Among various putative drought resistance mechanisms, the ability of plant to extract water from deeper soil profiles by growing deeper root systems is one the most relevant traits that directly influences yield under drought stress [27].

**Table 1:** Root traits of contrasting rice near-isogenic lines (NILS) grown in PVC pipes in greenhouse under stress and control conditions

		near-isogenic lines					
		
Trait	Condition	IR77298-14-1-2-B			IR77298-5-6-B		
			
		10	13	*diff*	18	11	*diff*
Maximum root length (cm)	well-water	51.50 ± 3.71	42.75 ± 3.71	ns	50.25 ± 3.71	39.00 ± 3.71	*
	drought stress	90.00 ± 4.65	79.75 ± 4.65	ns	75.00 ± 4.65	71.25 ± 4.65	ns
							
No. of roots per plants	well-water	231.13 ± 29.0	164.00 ± 29.0	ns	216.00 ± 29.0	164.13 ± 29.0	ns
	drought stress	115.50 ± 8.01	94.38 ± 8.01	*	112.75 ± 8.01	110.88 ± 8.01	ns
							
Root thickness (mm)	well-water	0.74 ± 0.05	0.76 ± 0.05	ns	0.82 ± 0.05	0.79 ± 0.05	ns
	drought stress	1.05 ± 0.07	0.85 ± 0.07	*	0.67 ± 0.07	0.74 ± 0.07	ns
							
Root volume (cm^3^)	well-water	6.03 ± 2.09	3.73 ± 2.41	ns	5.76 ± 2.09	3.26 ± 2.09	ns
	drought stress	2.84 ± 0.51	1.86 ± 0.51	ns	1.88 ± 0.51	1.76 ± 0.51	ns
							
Root dry weight (g)	well-water	3.20 ± 0.68	1.73 ± 0.78	ns	2.86 ± 0.68	1.51 ± 0.68	ns
	drought stress	1.44 ± 0.18	0.82 ± 0.18	**	0.98 ± 0.18	1.14 ± 0.18	ns

*diff*: shows the significant difference of pair of NIL, where *, ** Significant at p < 0.05 and 0.01 level respectively; ns non-significant,

well-water: is control condition with FTSW = 1.0, and drought stress: is drought stress treatment with FTSW = 0.2,

10: IR77298-14-1-2-B tolerant NIL i.e. IR77298-14-1-2-B-10; 13: IR77298-14-1-2-B susceptible NIL i.e. IR77298-14-1-2-B-13,

In this table, "drought stress" is 0.2 FTSW and "well-water" is control treatment. Data are mean of four replications.

### Microarray expression profiling

To gain a better understanding of the mechanism underlying the drought tolerance in roots, we applied a 4 × 44 K microarray system (platform no. GPL7252 is available at NCBI GEO) to examine expression profiles in roots of two pairs of NILs in the non-stressed and two drought stress regimes at reproductive stage.

The numerical comparison of DEGs obtained from three biological replications of microarray experiments in roots of NILs under different drought stress treatments is shown in Table 2. Overall, a total of 24027 transcripts out of 43494 (55%) were either up or down-regulated in at least two situations under drought stress treatments among rice genotypes. Differentiation of expression patterns of root tissue in different rice genotypes indicated that the number of DEGs under 0.2 FTSW was higher than 0.5 FTSW. A similar result was reported earlier indicating that a greater number of DEGs was found in roots of rice under high-osmotic treatment than low-osmotic treatment [28]. The results also indicated there was a relatively large set of genes that were commonly expressed in drought stress treatments. There were 5760 and 3846 genes commonly induced in response to 0.2 and 0.5FTSW; and 4815 and 3794 genes commonly repressed at 0.2 and 0.5 FTSW, respectively (Additional file 1). Response directions (up- or down-regulated transcripts) of individual DEGs by drought stress treatment were compared among the NILs (Table 2). In total, changes in number of DEGs between stress treatments and untreated plants (both up- and down-regulated) were highest for IR77298-14-1-2-B-10. As the level of drought stress increased, the number of DEGs also increased, suggesting that more genes were affected by increasing drought stress severity. Thus, despite their common genetic background as backcross progeny from Aday Sel × IR64, the two pairs of NILs showed distinctive differences in their gene expression profiles in response to drought.

**Table 2:** The number of up- and down-regulated genes in roots of rice genotypes under different drought stress treatments

		Genotypes					
			
drought stress	DEG		IR77298-14-1-2-B		IR77298-5-6-B		common
				
(FTSW)		IR64	10	13	18	11	
	Up	9686	10,232	9559	9262	8956	5760
0.2	Down	7218	7586	7532	7340	7400	4815
	total	16,904	17,818	17,091	16,602	16,356	10,755
							
	Up	7780	8675	7975	8575	8488	3846
0.5	Down	6662	7539	7067	7348	7533	3794
	total	14,442	16,214	15,042	15,923	16,021	7640

DEG: differentially expressed genes; Up: up-regulated; and Down: down-regulated

10: IR77298-14-1-2-B tolerant NIL i.e. IR77298-14-1-2-B-10; 13: IR77298-14-1-2-B susceptible NIL i.e. IR77298-14-1-2-B-13,

18: IR77298-5-6-B tolerant NIL i.e. IR77298-5-6-B-18; 11: IR77298-5-6-B susceptible NIL i.e. IR77298-5-6-B-11.

0.2 and 0.5 FTSW are severe and mild drought stress treatments

### Confirmation of microarray data by qRT-PCR

**Figure 1:**
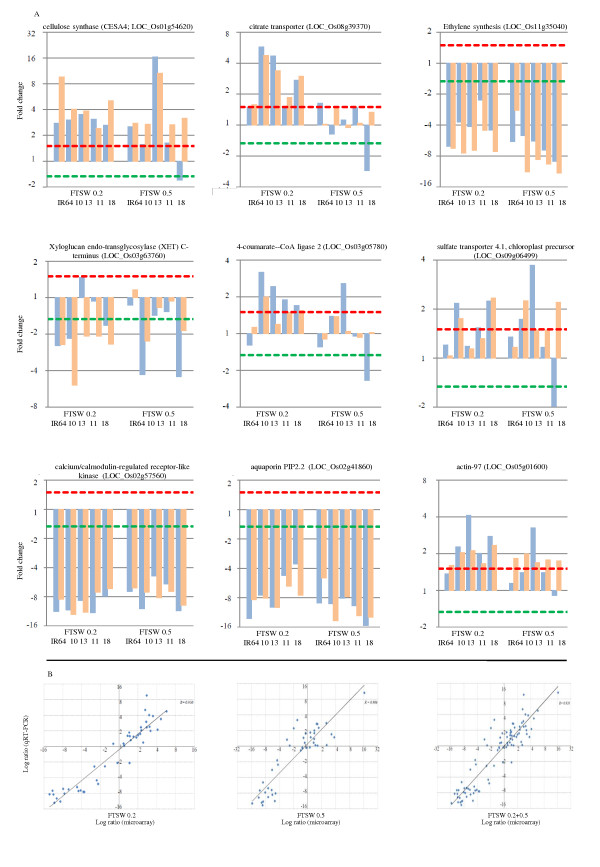
**Confirmation of expression profiles by qRT-PCR experiment with 9 selected DEGs compared to microarray data in roots of rice NILs under 3 drought stress treatments with three biological replications for each stress condition**. (A) Indicates comparison of microarray expression patterns of 9 selected DEGs from different functional categories with qRT-PCR data. In the histograms: × axis shows different rice genotypes used in this experiment i.e IR64 and four NILs including 10 = IR77298-14-1-2-B-10, 13 = IR77298-14-1-2-B-13, 11 = IR77298-5-6-B-11, and 18 = IR77298-5-6-B-18, severity of drought stress: FTSW0.2 indicates 20 percent of fraction of transpirable soil water which is considered as severe drought stress treatment, and FTSW0.5 indicates 50 percent of fraction of transpirable soil water which is considered as mild drought stress treatment., and Y axis representing fold changes in gene expression were transformed to log_2 _scale, dotted lines indicate 1.5 fold or 1/1.5 fold, blue bar indicates the median of three qRT-PCR replicates, orange bar indicates the results of the median of three replications of microarray experiments. (B) Correlation analysis of the ratio of differentially expression level from microarray experiment to that from qRT-PCR at different drought stress treatments which are in good agreement with each other. The microarray data log_2_-values (*X*-axis) were plotted against the qRT-PCR log_2_-values (*Y*-axis). FTSW0.2 indicates 20 percent of fraction of transpirable soil water which is considered as severe drought stress treatment; FTSW0.5 indicates 50 percent of fraction of transpirable soil water which is considered as mild drought stress treatment and FTSW0.2+0.5 representing overall drought stress treatments.

To assess the accuracy of microarray data, we selected 9 DEGs such as cellulose synthase (CESA4): LOC_Os01g54620 and others based on the biological importance as shown in Additional file 2 from the expression profiles of the genes that show up- or down-regulation among four NILs and IR64 for all drought stress treatments as well as control condition, while faintly changing genes were neglected. Then, we tested the similarity between gene expression identified by microarray and those by qRT-PCR (Figure 1). We observed that microarray and qRT-PCR data, which were calculated based on the median of three repeats, showed good correlation at different water stress treatments and overall water stress conditions (*r *= 0.906 ~ 0.950) and most cases of up/down-regulated expression of genes identified by microarray were also detected by qRT-PCR. Hence, the results suggesting that the DEGs identified through microarrays confirm actual differences between drought-stressed and non-stressed rice genotypes.

### Differentially-expressed genes in drought tolerant NILs

The analysis of the genes found exclusively in the tolerant genotypes is of interest to identify putative genes associated with drought tolerance. The identification of DEGs in the tolerant genotypes could reveal the metabolic and cellular processes that are ultimately responsible for stress tolerance [29]. In this respect, we considered specific DEGs in tolerant NILs compared to their susceptible sister NIL and IR64, the susceptible recurrent parent. A total of 1264 and 780 genes in IR77298-14-1-2-B-10; and 859 and 739 genes in IR77298-5-6-B-18 were specifically up- and down-regulated at 0.2 FTSW, in which 39 and 23 genes were expressed reversely in IR77298-14-1-2-B-13 and IR64, and 38 and 146 transcripts in IR77298-5-6-B-11 and IR64, respectively (Additional file 3). Many of these identified specific DEGs in tolerant NILs were shown previously to be involved in abiotic stress response [23,30]. These sets of DEGs were subjected to further analysis to investigate the biological functions of the DEGs in response to drought stress.

### Gene enrichment analysis for differentially expressed genes in NILs

**Figure 2:**
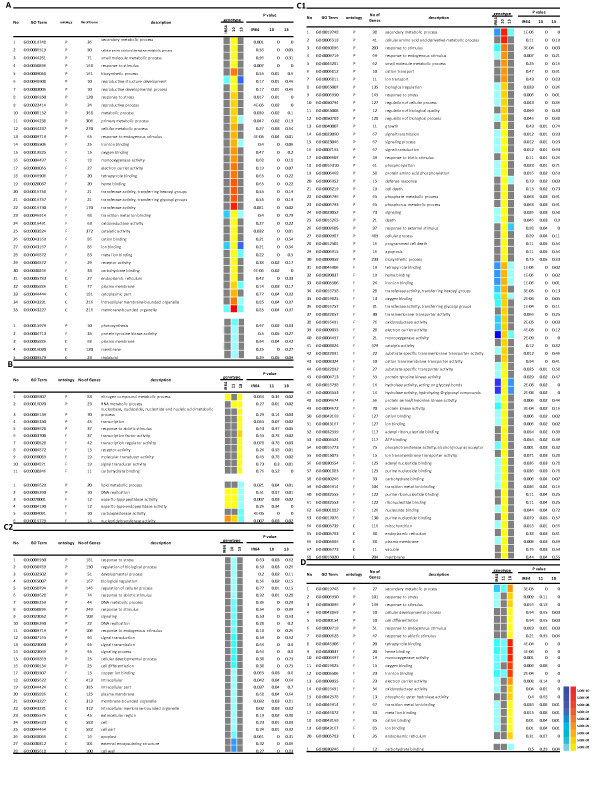
**Gene ontology enrichment analysis of genes specifically expressed in tolerant NILs of rice in response to water stress treatments in root tissue**. This figure shows a colourful model of parametric analysis of gene set enrichment (PAGE) using agriGO web-based tool of tolerant NILs versus their susceptible counterparts in response to different drought stress treatments (0.2 and 0.5 FTSW) applied in this study. In the figure, the information includes: GO term, ontology including three GO categories namely biological process (P), molecular function (F) and cellular component (C), number of annotated genes in each GO term, GO description, a simple colourful model in which red colour system means up regulated and blue means down regulated, and adjusted P value (FDR), respectively. In this figure also genotype descriptions are: IR = IR64, 10 = IR77298-14-1-2-B-10, 13 = IR77298-14-1-2-B-13, 11 = IR77298-5-6-B-11, and 18 = IR77298-5-6-B-18 (A) specifically expressed DEGs in IR77298-14-1-2-B-10 at 0.2 FTSW, (B) specifically expressed DEGs in IR77298-5-6-B-18 at 0.2 FTSW, (C1) specifically activated DEGs in IR77298-14-1-2-B-10 at 0.5 FTSW, (C2) specifically repressed DEGs in IR77298-14-1-2-B-10 at 0.5 FTSW, (D) specifically expressed DEGs in IR77298-5-6-B-18 at 0.5 FTSW, The coloured blocks represent the level of expression of up-/down-regulation of each term at a certain drought stress. The yellow-to-red, cyan-to-blue and grayscale represent the term is activated, repressed and non-significant, respectively.

Gene Ontology (GO) terms are widely applied to understand biological significance of microarray differential gene expression data [31]. The specific DEGs in tolerant NILs at two drought stress treatments were analysed for GO category enrichment using agriGO [31]. Figure 2 includes the GO categories and enrichment analysis for the specific DEGs of two pairs of NILs over drought stress treatments. For up-regulated genes in IR77298-14-1-2-B-10, as for biological process, there were 13 significant enriched GO terms and the most significant GO terms were "secondary metabolic process" (GO:0019748), "cellular amino acid and derivative metabolic process" (GO:0006519), "small molecule metabolic process" (GO:0044281), and "response to stimulus" (GO:0050896).

As for molecular functions the up-regulated genes belong to 17 significantly enriched GO terms that terms of "iron ion binding" (GO:0005506), "oxygen binding" (GO:0019825), "monooxygenase activity" (GO:0004497), "electron carrier activity" (GO:0009055), "tetrapyrrole binding" (GO:0046906), and "heme binding" (GO:0020037) were the important significant enriched GO. The GO terms of endoplasmic reticulum (GO:0005783) was the most important significant term for cellular components.

Among specifically repressed genes in IR77298-14-1-2-B-10 at 0.2FTSW, there were five significant GO for: a) biological process: "photosynthesis" (GO:0015979); b) molecular function: "protein tyrosine kinase activity" (GO:0004713); and C) cellular component: "thylakoid" (GO:0009579), "membrane" (GO:0016020), and "plasma membrane" (GO:0005886). In tolerant NIL IR77298-5-6-B-18, for up-regulated genes at 0.2FTSW, the important GO term for biological process was "nitrogen compound metabolic process" (GO:0006807), and as for molecular functions, three GO terms of "transcription factor activity" (GO:0003700), "transcription regulator activity" (GO:0030528), and "receptor activity" (GO:0004872) demonstrated significant enrichment. We also found that for specific repressed genes in this tolerant NIL, they were classified into two significant enriched GO terms for: a) biological process including "DNA replication" (GO:0006260) and "lipid metabolic process" (GO:0006629); and b) molecular functions such as "nucleotidyltransferase activity" (GO:0016779) and "carboxylesterase activity" (GO:0004091).

On the other hand, in mild drought stress condition, we found that the induced genes in IR77298-14-1-2-B-10 were classified into 68 significant enriched GO terms. Some of these significant GO terms like "growth" (GO:0040007), "death" (GO:0016265), "cation transport" (GO:0006812), "ion transport" (GO:0006811), "defense response" (GO:0006952), "programmed cell death" (GO:0012501), "signaling" (GO:0023052), "signaling process" (GO:0023046), and "cell death" (GO:0008219) were specifically enriched GO terms at mild drought stress condition. The enriched GO terms for repressed genes in IR77298-14-1-2-B-10, were classified into 16 significantly enriched GO terms of biological process such as "cell differentiation" (GO:0030154) and " cellular developmental process" (GO:0048869). As for molecular function the significant term was "copper ion biding" (GO:0005507), and for cellular component the GO terms of "cell wall" (GO:0005618) and "external encapsulating structure" (GO:0030312) were more significant. GO enrichment analysis suggests that higher tolerance to drought in IR77298-14-1-2-B-10 is probably attributable to significant up-regulation of transport systems, signalling networks and defence components. On the other hand, IR77298-5-6-B-18 showed moderately tolerance to drought with a significant up-regulation of regulatory networks and amino acid metabolism. Several reports indicated many transcripts encoding mitochondrial and endoplasmic-reticulum proteins like cytochrome P450 gene families, the largest category was related to oxidative stress enzymes which mainly activated in IR77298-14-1-2-B-10, including iron ion binding (GO:0005506), oxygen binding (GO:0019825), monooxygenase activity (GO:0004497), electron carrier activity (GO:0009055) were elevated during a combination of drought and heat stress in *Arabidopsis*[32], various metabolic processes and stress tolerance [33], and long term drought stress in rice [2]. Overall the GO terms enrichment analysis suggests that different drought response strategies are used to achieve drought tolerance as manifested in the two tolerant NILs.

### Drought-responsive biological pathway analysis in roots of tolerant NILs

**Figure 3:**
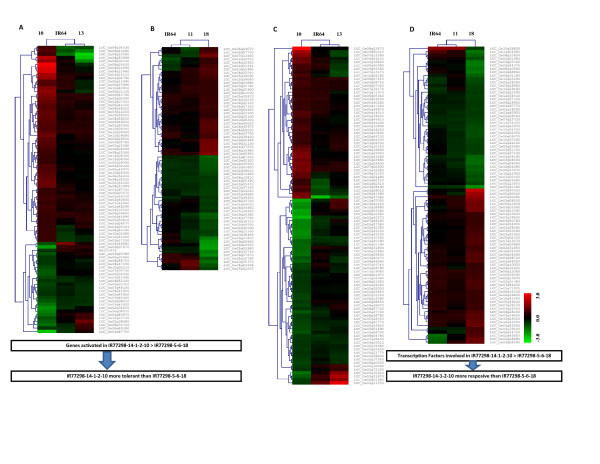
**General view of genes specifically expressed in two tolerant lines compared to their susceptible counterparts under severe drought stress condition**. In this figure: (A) indicates transcripts specifically expressed in IR77298-14-1-2-B-10, and (B) indicates transcripts specifically expressed in IR77298-5-6-B-18, from seven functional categories including cell growth, hormone biosynthesis, cellular transports, amino acid metabolism, reactive oxygen species (ROS), signaling and stress-regulated genes and carbohydrate metabolism. (C) and (D) indicate transcripts related to transcription factor families which are specifically expressed in two tolerant lines compared to their susceptible counterparts. Gene identifiers correspond to the each transcript are from MSU version 6.1 of Rice Oligoarray from Rice Genome Annotation Project (RGAP) 6.1 (http://rice.plantbiology.msu.edu/). A fold change ≥ 1.5 is shown in red (up-regulated), a fold change ≤ -1.5 is shown in green (down-regulated), and no change in black (FDR<0.05).

The DEGs in two tolerant NILs versus their susceptible counterparts were further analysed according to the various biological functions, which play important roles in drought stress tolerance. The functional categories were assembled from metabolic and signalling pathways available in different databases and in the literature. A detailed comparison of different NILs for DEGs in different functional categories is shown in Additional File 4. We also performed cluster analysis of genes specifically expressed in two tolerant lines IR77298-14-1-2-B-10 and IR77298-5-6-B-18 for these functional categories (Figure 3). There were obvious differences between the two tolerant lines. A greater number of genes was activated and more transcription factor gene families were differentially expressed in IR77298-14-1-2-B-10 compared to IR77298-5-6-B-18, further suggesting that the drought tolerance observed in the two tolerant lines is mediated by different mechanisms. Below, we focus on cases where the expression patterns bear relevance to the contrasting phenotypes observed in the NILs.

### Cell growth systems

Cell expansion in roots is crucial during drought stress. Expansion requires the coordinated activities of many cell processes [34]. In this study, cell wall-related genes were mostly down-regulated in roots of different rice genotypes under 0.2 FTSW at reproductive stage, while the number of up-regulated genes at 0.5 FTSW was higher in the same tissue and genotypes (Additional File 4). Similar results indicate that plant roots may continue growing in mild drought stress conditions [35]. These results indicate that severe drought stress seriously affected cell expansion in the roots of almost all rice genotypes at reproductive stage. While in the two tolerant NILs some genes were specifically up-regulated (Additional file 5). For instance, three genes involved in cell wall biosynthesis including two cellulose synthase like family-C (*CSLC-1*; LOC_Os01g56130) and -E (*CSLE-6*; LOC_Os09g30130) and one xyloglucan fucosyltransferase (*XG_FTase*; LOC_Os02g52640) were activated in IR77298-14-1-2-B-10. In the case of tolerant line IR77298-5-6-B-18, also three genes of *CSLA-9 *(LOC_Os06g42020), and *CSLC2 *(LOC_Os09g25900) and an *XG_FTase *(LOC_Os06g10980), were also specifically activated at 0.2FTSW. Several studies reported that members of the *CslA *subfamily encoding (1,4)-β-D-mannan synthases, and the *CslC *group are believed to encode an enzyme that directs the synthesis of the (1,4)-β-D-glucan which is considered as the backbone of xyloglucans [36-38]. Low water potential was shown to increase xyloglucan activity in maize roots, which was ascribed to the necessity of promoting root growth under these conditions [39]. Our results suggest that activation of these genes in root tips of tolerant rice under drought stress resulted in enhanced root growth and elongation. This is consistent with the observation that the tolerant lines have greater root development than the sensitive lines [A.Henry, Personal communications].

### Hormone biosynthesis

Many genes involved in hormone biosynthesis such as those related to abscisic acid (ABA), auxins, gibberellins and ethylene were found to be differentially expressed under severe drought stress (0.2 FTSW). In general, transcripts involved in hormone biosynthesis except for gibberellin were up-regulated under both drought stress treatments (Additional File 4). We found that genes related to ABA biosynthesis were constitutively activated in all rice genotypes. The plant hormone ABA plays a central role in many aspects of response to various stress signals [35], drought and high salinity-tolerance mechanisms [40]. One gene encoding beta-carotene hydroxylase (LOC_Os03g03370) was activated similarly in all lines both at 0.2 and 0.5 FTSW (Additional file 6). It was reported that overexpression of β-carotene hydroxylase enhances stress tolerance in *Arabidopsis *[41]. Overexpression of 9*-cis *epoxycarotenoid dioxygenase (*NCED*) including *OsNCED2 *(LOC_Os12g42280) and *OsNCED3 *(LOC_Os03g44380), key enzymes of ABA biosynthesis, was also observed in rice genotypes in this study. In *Arabidopsis*, *At-NCED3 *was strongly induced by dehydration and high salinity and its overexpression improved dehydration stress tolerance in transgenic plants [29,30].

We also found that genes involved in gibberellin biosynthesis were mostly down-regulated in response to drought stress treatments in different NILs (Additional file 6). It was shown that drought stress markedly increased ABA accumulation in rice grains and substantially decreased grain GA content [42]. Hence, repression of GA biosynthesis could be mediated through the activation of ABA biosynthesis in the root tissue of tolerant lines.

### Cellular transport systems

Plant cells respond to a wide variety of stimuli including biotic and abiotic stresses through development of different molecular transport systems. In this category, the number of DEGs in IR77298-14-1-2-B-10 was higher than other lines under two drought stress conditions (Additional file 4). We found that among DEGs, seven ion transports genes encoding glutamate receptor, nucleobase-ascorbate transporters, and oxidoreductases were specifically up-regulated in IR77298-14-1-2-B-10, and seven ion transporters gene in IR77298-5-6-B-18 under severe drought stress. In mild stress, three genes including a major intrinsic proteins (*MIP-TIP*; LOC_Os01g10600), *OEP21 *(LOC_Os02g58550), and oxidoreductase (LOC_Os10g02380) were activated in IR77298-14-1-2-B-10, and one nucleobase-ascorbate transporter (LOC_Os09g15170) in IR77298-5-6-B-18 (Additional file 7). In rice, the expression of *OsTIP1;1 *increased under drought, salt stress and exogenous ABA [43]. Hence, higher water uptake, water and ion transports in root tissues in tolerant NILs can be attributed to the over-expression of the above mentioned genes. In this study we observed that several genes related to pumps, secondary transporters were activated and/or repressed in two tolerant NILs compared with their susceptible counterparts under severe water stresses. This results indicate that a greater number of genes encoding ion transports including Na^+^, K^+^, and Ca^2+ ^were activated in highly tolerant NIL(IR77298-14-1-2-B-10) than in moderately tolerant (IR77298-5-6-B-18), indicating a potential role played by these genes in signal transduction in drought stress.

### Amino acid metabolism

Amino acids serve as precursors for a large array of metabolites with multiple functions in plant growth and response to various stresses. Proline metabolism is an important process in plant response to various abiotic stresses. Proline accumulation in roots of tolerant cultivars of rice starts earlier after the initiation of the stress treatment and was significantly more than their leaves [44]. The expression patterns of genes encoding enzymes involved in proline/arginine metabolism showed both up and down-regulation in the two tolerant NILs as compared with the susceptible lines. The number of activated genes for proline metabolism in IR77298-14-1-2-B-10 was higher than other lines under 0.2FTSW, while IR77298-5-6-B-18 showed more activated genes for proline metabolism under mild drought stress (Additional file 4). Some key genes encoding acetylglutamate kinase (LOC_Os04g46460), aldehyde dehydrogenase (family 2 and 3 of ALDH), glutamate synthase (LOC_Os03g50490), which are involved in reduction of glutamate to Δ^1^-pyrroline-5-carboxylate [45], specifically activated in IR77298-14-1-2-B-10 at 0.2FTSW (Additional file 8). We also observed that genes encoding Δ^1^-pyrroline-5-carboxylate synthetase (*P5CS*; LOC_Os05g38150) and arginase (LOC_Os04g01590) were the most over-expressed genes in IR77298-14-1-2-B-10 among rice genotypes under severe stress. The *P5CS *catalyzes the conversion of glutamate to Δ^1^-pyrroline- 5-carboxylate and arginase converts arginine to ornithine [45].

### Protection from oxidative damage

Reactive oxygen species (ROS) control many different processes in plants. However, excessive levels of ROS produce oxidative stress and inhibit plant root growth. ROS such as superoxide (O**^•-;^_2_**) and hydroxyl radicals (**^•^**OH) accumulate under stress conditions and need to be kept under control to preserve the integrity of cellular macromolecules [46]. In this study major ROS-scavenging enzymes and antioxidants of plants including superoxide dismutase (SOD), ascorbate peroxidase (APX), glutathione peroxidase (GPX) and peroxiredoxin (PrxR), glutaredoxins, glutathione were activated in response to drought stress treatments, and in greater number in IR77298-14-1-2-B-10 than in other lines (Additional file 4). These ROS scavengers provide the cells with an efficient machinery for detoxifying O**^•-;^_2 _**and H_2_O_2 _and constitute the first line of defense against ROS [47].

We also observed that the expression level of genes in superoxide dismutase (SOD) family was higher in tolerant NILs. Hence, reducing superoxide (O**^•-;^_2_**) could be higher in tolerant NILs than in susceptible lines (Additional file 9). SOD converts hydrogen superoxide into hydrogen peroxide [48]. A previous study showed that in rice, drought stress increased SOD activity [23].

In this study repression of a gene encoding an OsGrx_C2.1 in IR77298-5-6-B-18 at 0.2 FTSW and one gene (*OsGrx_C15*) in IR77298-14-1-2-B-10 at 0.5 FTSW were observed (Additional file 9). The OsGrx_C15 was reported to be repressed in response to *Magnaporthe grisea *infection in roots of rice [49].

We found that the defense mechanisms in IR77298-14-1-2-B-10 are more activated than the other NILs.

### Signaling and other abiotic stress regulated genes

In this biological category expression profiles of important genes involved in stress signaling systems and other stress regulated genes like chaperons (including dehydrins and late embryogenesis abundant, LEA) and some other important families were analysed (Additional file 4). Expression profile indicates that genes involved in this category mostly activated in response to drought stress treatments in different NILs, and the number of DEGs was higher at 0.2 FTSW, and a majority of them (38.9%) were similarly activated as compared to 0.5 FTSW (18.2%). Several study reported that under drought stress many chaperons including Hsp70, Hsf8-like [23], HSP70/DNAK, putative ATP-dependent Clp protease ATP-binding subunit [50], and also dehydrins and LEA gene members were activated in response to dehydration and drought stress [51,52] in rice cultivars.

Genes associated with signal transduction such as ABA responsive, calcium dependent protein kinases (CDPKs), calcineurin B-like protein-interacting protein kinases (CIPKs), calmodulin (CML) and calmodulin-related calcium sensor proteins, and receptor-like cytoplasmic kinases (RLCKs) were both up and down-regulated in response to drought stress treatments in different NILs (Additional file 4). In case of ABA responsive genes, a greater number of DEGs commonly activated in all lines, with higher level of expression in tolerant NILs, in response to severe drought stress treatment than mild stress (Additional file 10). Results indicate that a serine/threonine-protein kinase receptor precursor (LOC_Os04g34330) was specifically activated in IR77298-14-1-2-B-10 under 0.2FTSW. The transcript serine/threonine-protein kinase receptor precursor (LOC_Os04g34330) was reported to be highly responsive to ABA under drought stress in roots of rice [53].

In CDPKs family, which plays an essential role in plant Ca^2+^-mediated signal transduction [54], results indicate that more genes were activated in tolerant line IR77298-14-1-2-B-10 compared to IR77298-5-6-B-18 under severe drought stress (Additional file 4). One transcript of *OsCPK15 *was specifically activated in IR77298-14-1-2-B-10 under severe drought stress; while under mild stress *OsCPK28 *was activated (Additional file 10). It was reported that *OsCPK15 *was induced in response to drought stress in roots of rice, and salt stress [54].

We found that although the induction of genes involved in this category is a common response in roots of rice lines at reproductive stage, a greater number of genes encoding chaperons, ABA responsive genes, CDPK, calmodulins and RLCK genes were specifically activated in IR77298-14-1-2-B-10 under severe drought stress. Furthermore, a greater number of CIPK family members were specifically activated in IR77298-5-6-B-18. Under mild drought stress more genes related to chaperons, CIPKs were uniquely activated in IR77298-14-1-2-B-10 and a higher number of CDPKs, calmodulins and RLCKs were uniquely activated in IR77298-5-6-B-18.

### Transcription factors

Transcription factors (TFs) regulate gene expression in response to environmental and physiological signals. In this study, 1461 (62.5%) out of 2336 genes from TF gene families were differentially expressed in different NILs in response to drought stress treatments. The number of TF genes differentially expressed under severe drought stress was greater than mild drought stress (Additional file 4). Among DEGs for TF genes about 50% (287 up- and 436 down-regulated) and 35% (193 up- and 323 down-regulated) were similarly expressed at 0.2 and 0.5 FTSW, respectively. In two tolerant NILs, 13 TF genes from AP2-EREBP, bHLH, C2H2, GRAS, HB, LOB, MYB-related and OFP families were similarly activated, and eight gene members from ARR-B, CCAAT, FAR1, MADS, Orphans, SNF2 and Trihelix families were commonly repressed in response to severe drought stress treatment. Under mild drought stress 16 genes related to ARF, BBR/BPC, bHLH, C2C2-CO-like, C2C2-Dof, C3H, G2-like, GeBP, GRAS, HB, NAC, SET and WRKY families and six TF transcripts from bHLH, C2C2-Dof, C2H2, FHA and OFP were similarly up- and down-regulated (Additional file 11). In IR77298-14-1-2-B-10, 42 and 49 TF genes were specifically up- and down-regulated, respectively, in response to severe drought stress. The activated TF genes in highly tolerant NIL mostly belong to AP2-EREBP (7), bHLH (3), bZIP (2), C2H2(5), FHA(2), GNAT(4), NAC (1), and WRKY (2) families. In moderately tolerant NIL, 39 TF genes were specifically activated and 36 repressed. These activated TF genes were mostly from AP2-EREBP (5), bHLH (3), C2H2 (2), FAR1 (2), NAC (1) and WRKY (4) (Table 3). Several studies reported that AP2-EREBP, C2C2, CCAAT, bZIP, WRKY, NAC, bHLH families play an important role in drought tolerance in rice [29,55,56]. Overall, IR77298-14-1-2-B-10 showed the greatest number of responsive TF gene families under severe drought stress while a greater number of TF genes were activated in IR77298-5-6-B-18 under mild stress, suggesting that IR77298-14-1-2-B-10 is the more responsive rice genotype under severe drought stress treatment, where IR77298-5-6-B-18 is responsive to mild stress treatment.

**Table 3:** The number of differentially expressed genes in transcription factor families in tolerant NILs in response to different drought stress treatments.

	Number of genes															
	
TF Family	10				18				10vs13IR				18vs11IR			
				
	0.2		0.5		0.2		0.5		0.2		0.5		0.2		0.5	
								
	Up	down	Up	down	Up	down	Up	down	Up	down	Up	down	Up	down	Up	down
ABI3VP1	12	10	12	9	8	9	10	12	1	1	1	1	1	0	1	4
Alfin-like	0	3	0	3	0	1	0	3	0	0	0	0	0	0	0	0
AP2-EREBP	25	73	27	60	25	67	25	65	7	3	2	6	5	0	1	6
ARF	10	3	11	4	11	3	10	4	0	0	2	1	0	0	2	0
ARID	2	1	1	0	1	1	0	2	0	0	0	0	0	0	0	1
ARR-B	1	1	1	2	1	2	2	1	0	1	0	0	0	1	0	0
AUX/IAA	4	19	5	16	1	17	4	17	0	0	0	1	0	1	0	0
BBR/BPC	2	0	2	0	1	0	2	0	0	0	1	0	0	0	1	0
BES1	1	1	1	0	1	1	1	1	0	0	0	0	0	0	0	1
bHLH	25	44	23	50	26	48	24	48	3	2	4	5	3	4	3	2
BSD	2	1	1	1	0	0	0	1	0	0	0	0	0	0	0	0
bZIP	22	32	18	26	20	30	20	25	1	2	2	2	1	1	2	2
C2C2-CO-like	10	3	11	2	9	3	10	1	1	0	3	0	1	0	1	0
C2C2-Dof	3	13	4	10	6	10	7	8	1	1	2	1	1	0	3	1
C2C2-GATA	7	7	7	5	7	8	5	7	0	0	2	2	0	0	0	0
C2C2-YABBY	0	1	0	1	0	1	0	2	0	0	0	0	0	0	0	0
C2H2	19	33	21	28	16	31	17	28	5	2	5	5	2	1	1	1
C3H	20	12	13	12	18	9	16	7	0	1	3	3	0	3	3	3
CAMTA	1	0	0	1	0	0	0	0	0	0	0	0	0	0	0	0
CCAAT	8	17	4	16	6	13	9	13	0	2	1	3	1	3	3	0
Coactivator p15	2	0	2	0	2	0	2	0	0	0	0	0	0	0	0	0
CPP	3	1	1	1	2	2	2	1	0	0	0	0	0	0	0	0
DBP	3	1	3	0	3	1	3	0	0	0	0	0	1	0	0	0
DDT	2	0	2	0	2	0	2	0	0	0	0	0	0	0	0	0
E2F-DP	2	2	1	1	2	2	1	1	0	0	0	0	0	0	0	0
EIL	5	1	4	1	4	0	4	0	1	0	0	0	0	0	0	0
FAR1	17	3	11	4	16	3	13	1	1	1	2	1	2	1	1	1
FHA	10	4	6	3	5	3	5	2	1	2	3	1	0	0	0	1
G2-like	11	12	11	9	9	13	10	9	2	0	4	0	0	1	2	0
GeBP	2	0	3	0	4	0	2	0	0	0	2	0	0	0	2	0
GNAT	13	11	10	8	9	9	9	8	4	1	2	1	0	0	1	1
GRAS	14	16	8	14	12	15	10	19	2	2	3	1	2	2	1	3
GRF	3	4	3	4	3	4	3	4	0	0	0	3	0	0	0	0
HB	19	30	18	29	19	27	20	27	1	2	2	5	1	1	1	0
HMG	3	2	2	3	4	2	2	4	0	0	0	0	0	0	0	1
HSF	12	4	11	4	12	5	10	4	0	0	1	2	1	0	1	0
IWS1	1	0	0	0	0	0	0	0	0	0	0	0	0	0	0	0
Jumonji	1	5	0	6	0	6	1	6	0	0	0	0	0	1	0	0
LIM	1	1	1	1	1	1	1	1	0	0	0	0	0	0	0	0
LOB	5	9	6	10	6	9	2	10	1	0	0	1	2	0	0	0
LUG	2	1	2	1	2	1	2	1	0	0	0	0	0	1	0	0
MADS	7	16	4	13	4	14	6	12	1	3	0	3	0	2	2	1
MBF1	0	2	0	2	0	2	0	2	0	0	0	0	0	0	0	0
mTERF	10	3	7	1	9	3	6	0	0	0	1	0	0	0	0	0
MYB	22	35	22	32	21	39	20	38	0	1	4	5	0	0	1	4
MYB-related	17	19	13	23	11	19	13	21	1	1	4	1	1	1	3	2
NAC	15	44	20	32	15	44	19	30	1	1	3	3	1	1	3	1
OFP	2	15	2	14	1	14	2	15	2	2	1	5	1	0	0	2
Orphans	15	20	13	22	13	21	10	24	0	5	3	2	2	4	1	0
PHD	16	6	11	6	13	5	10	6	0	1	0	1	1	0	0	1
PLATZ	1	2	2	2	3	3	3	3	1	0	1	0	1	0	0	1
Pseudo ARR-B	3	0	3	0	2	0	3	0	0	0	0	0	0	0	0	0
RB	0	1	0	1	0	1	0	1	0	0	0	1	0	0	0	0
Rcd1-like	1	0	0	0	0	0	2	0	0	0	0	0	0	0	0	0
RWP-RK	2	1	2	3	2	1	1	1	0	0	1	0	0	0	0	0
S1Fa-like	0	0	0	1	0	1	0	0	0	0	0	0	0	0	0	0
SBP	4	5	3	6	6	6	5	6	0	0	0	0	0	0	0	1
SET	10	3	7	4	8	2	8	4	0	0	5	1	0	0	3	1
Sigma70-like	5	5	3	0	5	0	5	0	0	0	1	0	0	0	1	0
SNF2	11	5	6	4	10	4	7	4	0	1	0	1	0	1	0	1
SOH1	1	0	1	0	2	0	1	0	0	0	1	0	1	0	1	0
SRS	0	0	0	3	0	4	0	4	0	1	0	1	0	0	0	0
SWI/SNF-BAF60b	1	0	1	0	1	0	1	0	0	0	0	0	0	0	0	0
SWI/SNF-SWI3	0	1	0	1	1	1	0	0	0	1	0	0	0	1	0	0
TAZ	0	2	0	2	0	2	0	2	0	0	0	0	0	0	0	1
TCP	6	6	5	5	6	3	8	5	0	2	1	1	0	0	2	2
Tify	4	7	3	8	1	10	2	9	0	0	0	1	0	3	0	0
TRAF	6	8	5	10	5	9	7	11	1	0	2	1	1	1	3	0
Trihelix	5	6	5	4	6	3	7	3	0	3	2	1	1	1	1	0
TUB	4	4	4	4	3	4	5	2	1	1	2	2	0	0	0	0
WRKY	13	48	18	38	13	51	15	45	2	3	9	2	4	0	4	1
zf-HD	1	3	1	2	2	2	2	2	0	0	0	0	1	0	1	0

Total	487	648	428	588	437	625	434	593	42	49	88	77	39	36	56	47

In this table, 10 and 18 are two tolerant NILs (IR77298-14-1-2-B-10 and IR77298-5-6-B-18), 10vs13IR and 18vs11IR are comparisons of DEGs specifically expressed in tolerant NILs against their susceptible counterparts (IR77298-14-1-2-B-10 versus IR77298-14-1-2-B-13 and IR64; and IR77298-5-6-B-18 versus IR77298-5-6-B-11 and IR64), respectively. 0.2 and 0.5 are two FTSW treatments. Up = significantly up-regulated genes, down = significantly down-regulated gen

### Carbohydrate metabolism

In this study many genes involved in carbohydrate metabolism were found to be differentially expressed under drought stress treatments such as those related to glycolysis, citrate cycle (TCA), starch-sucrose, fructose-mannose metabolism. These DEGs were mostly activated in response to drought stress treatments in different NILs (Additional file 4). Changes in carbohydrate metabolism are typical physiological and biochemical response to stress. For instance, we found that four genes encoding 6-phosphofructokinase (LOC_Os01g09570), aldehyde dehydrogenases (LOC_Os06g15990 and LOC_Os11g08300) and hexokinase (LOC_Os01g53930) were specifically up-regulated in IR77298-14-1-2-B-10 under 0.2 FTSW (Additional file 12). This set of activated gene was reported to play important roles in glycolysis [57]. Results also indicated that genes involved in citrate cycle and starch-sucrose and ascorbate-aldarate metabolism were mostly activated in different NILs in response to drought stress treatments. This includes a variety of sucrose synthases, soluble starch synthases and starch branching enzymes. Genes involved in fructose-mannose, inositol-phosphate metabolisms were both up and down-regulated (Additional file 4). For starch and sucrose metabolism two genes encoding alpha-amylase isozyme C2 (LOC_Os06g49970) and hexokinase-1 (LOC_Os01g53930) were specifically up-regulated in IR77298-14-1-2-B-10 under severe stress treatment. Under mild stress treatment three genes encoding hexokinase-1 (LOC_Os01g53930), hexokinase-2 (LOC_Os05g44760) and UDP-glucose 6-dehydrogenase (LOC_Os12g25700) were activated in two tolerant lines, among them UDP-glucose 6-dehydrogenase was common to these tolerant lines (Additional file 12).

In the context of carbohydrate metabolism, a relatively large proportion of genes related to sucrose synthesis, glycolysis, TCA cycle, ascorbate and aldarate metabolism, and fructose mannose metabolism were activated in IR77298-14-1-2-B-10 compared to IR77298-5-6-B-18 under severe drought stress, suggesting that the tolerant NIL may adopt a strategy of reserving sufficient carbon sources and energy for the growth of lateral root and root hair [58], detoxification of acetaldehyde, and sugar sensing and signaling [29].

### Root-specific DEGs in different rice NILs

Drought stress leads to growth inhibition of leaves, whereas roots may continue to grow and send the stress signal to the shoot [17]. Hence, to identify some tissue-specific regulated genes under drought stress, we surveyed DEGs in root and leaf tissues for some biological categories such as cell growth systems, hormone biosynthesis, amino acid metabolism, transport systems and transcription factors. We found that a relatively large number of root-specific genes are involved in cell growth, hormones biosynthesis, amino acid metabolism, transport systems and transcription factors and etc in rice NILs (Additional file 13). Several reports indicated that the differences between root and leaf tissues under drought stress could be attributed to the activation of genes like expansin, cellulose synthase and xyloglucans families [34,36,37,46], which are involved in root growth under water-deficit. A higher level of ABA accumulation in roots of NILs was also observed, which plays a vital role in stress signalling from root to shoot [29,30,35,40]. Auxins related genes were specifically activated in roots, which may regulate lateral root formation [59]. Tolerant NILs showed a higher accumulation of proline in root, which is a possible indicator of the osmotic tolerance [45,46]. We also observed that cellular transports which play important roles in plant cells respond to various stimuli such as drought and salinity [43] are activated to a greater extent in roots of tolerant NILs as compared to leaf tissues. Many stress-response related TF genes such as bZIPs, AP2-EREBPs, EIL, HBs, were specifically expressed in root tissues of tolerant NILs in response to drought stress treatments [60]. Some of these TF genes like NAC (LOC_Os02g57650), SNFs (LOC_Os02g32570, LOC_Os04g47830), bZIP (LOC_Os09g13570) genes were specifically activated in root tissue. We observed that root-specific DEGs from different biological categories mostly were either specific to tolerant NILs. The level of expression of these genes was higher in two tolerant NILs compared to the susceptible NILs.

## Conclusions

Application of a new comprehensive 44K oligoarray platform together with a dry-down method enabled us to determine the gene expression profiles in roots of two pairs of NILs with contrasting yield performance under drought stress treatments at reproductive stage. Overall, across all rice genotypes, the number of DEGs is higher in response to severe drought stress than to mild drought stress, suggesting that more genes were affected by increasing drought stress. The number of commonly expressed genes among genotypes and treatments also was higher under severe stress. Hence, comparison of a pair of NILs with contrasting phenotypes can reveal important genes regulating drought tolerance. By comparing the expression patterns of NILs, we identified the important categories of genes, the expression of which can clearly differentiate the tolerance and susceptible genotypes.

Although the two pairs of NILs were derived from a common background, they appear to carry different mechanisms for tolerance to drought stress. As a connection between different biological pathways in two tolerant NILs, the earliest response to water deficit could be overexpression of genes encoding enzymes related to ABA synthesis, especially in IR77298-14-1-2-B-10. Differences in response mechanisms were also supported by the detailed changes in gene expression patterns under drought conditions. The regulatory effects of these genes together with key gene members of different functional categories should be studied in more details.

According to probe sets position, we found that genes specifically activated from different functional categories mostly located on chromosomes 1, 2, 4, 6, and 9 in IR77298-14-1-2-B-10; and 1, 3, 4 and 6 in IR77298-5-6-B-18 over drought stress treatments, as some of them previously reported. Hence, results of this study could be combined with QTL analysis to identify genes useful for rice breeding programs.

## Methods

### Plant Materials and Stress Conditions

Plant materials used in this study are two pairs of NILs contrasting for yield under drought stress and IR64. Among the NILs, one pair was derived from IR77298-14-1-2 family and the other from IR77298-5-6 family at IRRI [12]. IR77298-14-1-2 and IR77298-5-6 are tungro tolerant sister lines developed at IRRI by backcrossing Aday Sel. (a tungro tolerant variety from India) to IR64 [10], and these two lines were also found to be differing in drought tolerance [12]. Of the NIL pair from IR77298-14-1-2 family IR77298-14-1-2-B-10 was high-yielding (highly drought-tolerant) while IR77298-14-1-2-B-13 was low-yielding under stress (susceptible); similarly, from the IR77298-5-6 family, IR77298-5-6-B-18 was high yielding (moderately drought-tolerant) while and IR77298-5-6-B-11 was low-yielding under stress (highly susceptible). These four NILs possessed similar yield potential. Further, the contrasting NILs in a pair were at least 97% genetically similar [12].

Plant materials were grown in PVC pipe columns measuring 1.05 m and diameter 18 cm filled with 10 kgs of soil mix (2 soil: 1 sand), adequately fertilized and grown under controlled conditions (Initially grown in green house but shifted to phytotron before imposing the stress). Saturated soils in the pots were covered with white plastic covers, with an opening in the middle to facilitate planting. Feeder pipe was inserted for watering the pots. Five pre-germinated seeds transplanted per pot and later thinned to 2 plants at three leaf stage. The experimental design was a randomized complete block design (RCBD) with four replications.

All pots were irrigated twice daily to maintain the soil at saturation. The day before the start of progressive soil drying, soil in each pot was saturated. Stress was imposed by initiating soil dry down protocol starting 35 DAS and dried down until the pot reached targeted FTSW [25]. All the pots were allowed to dry down until there was no or negligible transpiration. The pots were weighed daily during the dry down to estimate the transpiration. The watering regime were (a) control, consisting of well-watered plants and soil kept saturated throughout the experiment, (b) drought stresses, including two drought stress conditions of 0.2 FTSW = 20% and 0.5 FTSW = 50%, no water was added back to the soil during dry down. Pots were maintained at targeted FTSW until harvest. At harvest data on maximum root length, root thickness, root volume, total root number, root dry weight and shoot dry weight were recorded.

### RNA extraction

Total RNA samples were extracted from 10 mm of roots tip of plant materials of all the treatments i.e. 1.0, 0.5 and 0.2 FTSW in three replications at reproductive stage by using an RNeasy Maxi kit (Qiagen). This part of root is the active growing region and is an important root part in responding to stress by way of root elongation [61]. The concentration and quality of microarray samples were examined by Nanodrop (Nanodrop ND-1000; Nanodrop Technologies) and BioAnalyzer (G2938A; Agilent Technologies). For the microarray experiments in this study, 60 independent RNA samples of roots were prepared.

### Microarray experiment and data analysis

In our study, the probe and array designs were performed through eArray version 4.5 supplied by Agilent Technologies https://earray.chem.agilent.com/earray/ and 43494 probes were selected for this custom array. Four sets of the 43494 probes (4x44K microarray formats) were blotted on a glass slide (25 x75 mm) at Agilent Technologies in three biological replications.

Cyanine 3 (Cy3)- or cyanine 5 (Cy5)-labelled cRNA samples were synthesized from 850 *ng *total RNA by using a Low Input RNA labelling kit (Agilent Technologies) according to the manufacturer's instructions. Transcriptome profiles specific to stressed plants were examined by direct comparison of transcription activities between stressed condition and non-stressed (control) plants on the same oligoarray. Hybridization solution was prepared with 825 *ng *each of Cy3- and Cy5-labelled cRNA preparations using an in situ Hybridization Kit Plus (Agilent Technologies). Hybridization and washing of microarray slides followed according to the manufacturer's protocols. After washing, slide image files were produced by a DNA microarray scanner (G2505B; Agilent Technologies).

Signal intensities of Cy3 and Cy5 were extracted from the image files and normalized to remove the dye effect in signal intensity by rank consistency and the LOWESS method, processed by Feature Extraction version 9.5 (Agilent Technologies). Signal intensities of all samples were transformed into log_2_-based numbers and normalized according to the quantile method for standardization among array slides by EXPANDER version 5.0 [62]. A gene was declared 'expressed' if the mean signal intensity of the gene was > 6 at least at one condition; otherwise, the gene was considered not expressed. Those genes were considered as differentially expressed (DEGs) which had (i) a log_2_-based ratio (stressed sample/control-nonstressed sample) >0.585 or, <-0.585, and (ii) the significance of changes in gene expression between two plants (*P*) ≤0.05 by a paired t-test (permutation, all; FDR collection, adjust Bonferroni method). Data processing was performed by using Multi experimental Viewer (MeV) version 4.5 [63]. GO enrichment analysis was performed on log_2_-based ratio of specifically expressed DEGs in tolerant NILs against their susceptible counterparts by using "agriGO" [31] through Parametric Analysis of Gene Set Enrichment (PAGE) method. The outputs of microarray analysis used in this study (series no. GSE30463) is available at NCBI GEO [64]. All data are MIAME compliant.

### qRT-PCR

To validate the results from the microarray experiment, 9 selected DEGs from different functional categories were analyzed using qRT- PCR.

Total RNA (160*ng*) was treated with DNase by the TURBO DNA-free Kit (Ambion) and reverse-transcribed by the iScriptTM cDNA Synthesis Kit (BIO-RAD). qRT-PCR reaction mixture was consist of the KAPA SYMR FAST qPCR Kit (KAPA BIOSYSTEMS) and 2 μl four times diluted cDNA reaction mixture in a final volume 20 μl with 200 nM of the gene specific primers as listed in Additional file 2. PCR reaction was performed with iCycler iQ (BIO-RAD) and the cycle as follows, denaturation at 95°C for 1 min, annealing and polymerization at 58°C for 20 seconds. Three biological repeats were made. As a reference gene for qRT-PCR, we have used UBC (Ubiquitin-conjugating enzyme E2) [65].

## Authors' contributions

AM carried out the microarray experiment for severe drought stress treatment, analyzed the data and drafted the manuscript. KS provided transcriptome data for mild drought stress treatment and contributed in data analysis. HK performed the qRT-PCR experiments. TA also contributed for setting up the qRT-PCR system. AH carried out some computational work. RV developed the NILs and carried out RNA extraction. RS supervised greenhouse experiments, participated in designing experiment especially the dry-down in green house and sampling of plant tissues, provided root physiological data used in this manuscript and contributed in manuscript revisions. AK provided plant materials, participated in experimental designing and manuscript revisions. HL participated in experiment designing and manuscript draft revision. SK designed the experiment, coordinated the program and provided all lab facilities for molecular analysis and helped to prepare the manuscript. All authors read and approved the final manuscript.

## Supplementary Material

Additional file 1 **Commonly expressed genes in roots of different rice genotypes under two drought stress treatments**. In this table, 0.2 FTSW indicates 20 percent of fraction of transpirable soil water which is considered as severe drought stress treatment, and 0.5 FTSW indicates 50 percent of fraction of transpirable soil water which is considered as mild drought stress treatment. 10 = IR77298-14-1-2-B-10, 13 = IR77298-14-1-2-B-13, 11 = IR77298-5-6-B-11, and 18 = IR77298-5-6-B-18. A log**_2_**ratio > 0.585 is shown as up-regulated gene, A log**_2_**ratio <-0.585 is shown as down-regulated gene with an adjusted P value (FDR) <0.05. Up = significantly up-regulated genes, down = significantly down-regulated genes. Click here for file

Additional file 2 **Selected genes and corresponding primer sequences used for qRT-PCR**. Click here for file

Additional file 3 **The significant genes specifically expressed in two tolerant NILs under two drought stress treatments**. In this table, 0.2 FTSW indicates 20 percent of fraction of transpirable soil water which is considered as severe drought stress treatment, and 0.5 FTSW indicates 50 percent of fraction of transpirable soil water which is considered as mild drought stress treatment. 10 = IR77298-14-1-2-B-10, 13 = IR77298-14-1-2-B-13, 11 = IR77298-5-6-B-11, and 18 = IR77298-5-6-B-18. A log**_2_**ratio > 0.585 is shown as up-regulated gene, a log**_2_**ratio <-0.585 is shown as down-regulated gene with an adjusted P value (FDR) <0.05. Up = significantly up-regulated genes, down = significantly down-regulated genes. Click here for file

Additional file 4 **Changes in transcription of genes of different functional categories in roots of rice genotypes in response to drought stress treatments**. In this table, 10 = IR77298-14-1-2-B-10, 13 = IR77298-14-1-2-B-13, 11 = IR77298-5-6-B-11, and 18 = IR77298-5-6-B-18. 10vs18 is comparisons of DEGs commonly expressed in IR77298-14-1-2-B-10 versus IR77298-5-6-B-18, respectively. Common: ALL shows commonly expressed DEGs in two tolerant NILs as well as all genotypes over all drought stress treatments. Click here for file

Additional file 5 **Gene expression profiles related to cell growth category in roots of rice genotypes under two drought stress treatments**. In this table, 0.2 FTSW indicates 20 percent of fraction of transpirable soil water which is considered as severe drought stress treatment, and 0.5 FTSW indicates 50 percent of fraction of transpirable soil water which is considered as mild drought stress treatment. A transcript is considered as up-regulated, if log**_2_**-ratio >0.585 and down-regulated if log**_2_**-ratio <-0.585, and a 0.585≥log**_2_**-ratio≥-0.585 is considered as no change. 10: IR77298-14-1-2-B-10, 13: IR77298-14-1-2-B-13, 11: IR77298-5-6-B-11, and 18: IR77298-5-6-B-18. Click here for file

Additional file 6 **Gene expression profiles related to hormone biosynthesis category in roots of rice genotypes under two drought stress treatments**. In this table, 0.2 FTSW indicates 20 percent of fraction of transpirable soil water which is considered as severe drought stress treatment, and 0.5 FTSW indicates 50 percent of fraction of transpirable soil water which is considered as mild drought stress treatment. A transcript is considered as up-regulated, if log**_2_**-ratio >0.585 and down-regulated if log**_2_**-ratio <-0.585, and a 0.585≥log**_2_**-ratio≥-0.585 is considered as no change. 10: IR77298-14-1-2-B-10, 13: IR77298-14-1-2-B-13, 11: IR77298-5-6-B-11, and 18: IR77298-5-6-B-18. Click here for file

Additional file 7 **Gene expression profiles related to cellular transport category in roots of rice genotypes under two drought stress treatments**. In this table, 0.2 FTSW indicates 20 percent of fraction of transpirable soil water which is considered as severe drought stress treatment, and 0.5 FTSW indicates 50 percent of fraction of transpirable soil water which is considered as mild drought stress treatment. A transcript is considered as up-regulated, if log**_2_**-ratio >0.585 and down-regulated if log**_2_**-ratio <-0.585, and a 0.585≥log**_2_**-ratio≥-0.585 is considered as no change. 10: IR77298-14-1-2-B-10, 13: IR77298-14-1-2-B-13, 11: IR77298-5-6-B-11, and 18: IR77298-5-6-B-18. Click here for file

Additional file 8 **Gene expression profiles related to amino acid metabolism category in roots of rice genotypes under two drought stress treatments**. In this table, 0.2 FTSW indicates 20 percent of fraction of transpirable soil water which is considered as severe drought stress treatment, and 0.5 FTSW indicates 50 percent of fraction of transpirable soil water which is considered as mild drought stress treatment. A transcript is considered as up-regulated, if log**_2_**-ratio >0.585 and down-regulated if log**_2_**-ratio <-0.585, and a 0.585≥log**_2_**-ratio≥-0.585 is considered as no change. 10: IR77298-14-1-2-B-10, 13: IR77298-14-1-2-B-13, 11: IR77298-5-6-B-11, and 18: IR77298-5-6-B-18. Click here for file

Additional file 9 **Gene expression profiles related to reactive oxygen species (ROS) category in roots of rice genotypes under two drought stress treatments**. In this table, 0.2 FTSW indicates 20 percent of fraction of transpirable soil water which is considered as severe drought stress treatment, and 0.5 FTSW indicates 50 percent of fraction of transpirable soil water which is considered as mild drought stress treatment. A transcript is considered as up-regulated, if log**_2_**-ratio >0.585 and down-regulated if log**_2_**-ratio <-0.585, and a 0.585≥log**_2_**-ratio≥-0.585 is considered as no change. 10: IR77298-14-1-2-B-10, 13: IR77298-14-1-2-B-13, 11: IR77298-5-6-B-11, and 18: IR77298-5-6-B-18. Click here for file

Additional file 10 **Gene expression profiles related to signaling and stress related category in roots of rice genotypes under two drought stress treatments**. In this table, 0.2 FTSW indicates 20 percent of fraction of transpirable soil water which is considered as severe drought stress treatment, and 0.5 FTSW indicates 50 percent of fraction of transpirable soil water which is considered as mild drought stress treatment. A transcript is considered as up-regulated, if log**_2_**-ratio >0.585 and down-regulated if log**_2_**-ratio <-0.585, and a 0.585≥log**_2_**-ratio≥-0.585 is considered as no change. 10: IR77298-14-1-2-B-10, 13: IR77298-14-1-2-B-13, 11: IR77298-5-6-B-11, and 18: IR77298-5-6-B-18. Click here for file

Additional file 11 **Gene expression profiles related to transcription factors category in roots of rice genotypes under two drought stress treatments**. In this table, 0.2 FTSW indicates 20 percent of fraction of transpirable soil water which is considered as severe drought stress treatment, and 0.5 FTSW indicates 50 percent of fraction of transpirable soil water which is considered as mild drought stress treatment. A transcript is considered as up-regulated, if log**_2_**-ratio >0.585 and down-regulated if log**_2_**-ratio <-0.585, and a 0.585≥log**_2_**-ratio≥-0.585 is considered as no change. 10: IR77298-14-1-2-B-10, 13: IR77298-14-1-2-B-13, 11: IR77298-5-6-B-11, and 18: IR77298-5-6-B-18. Click here for file

Additional file 12 **Gene expression profiles related to carbohydrate metabolism category in roots of rice genotypes under two drought stress treatments**. In this table, 0.2 FTSW indicates 20 percent of fraction of transpirable soil water which is considered as severe drought stress treatment, and 0.5 FTSW indicates 50 percent of fraction of transpirable soil water which is considered as mild drought stress treatment. A transcript is considered as up-regulated, if log**_2_**-ratio >0.585 and down-regulated if log**_2_**-ratio <-0.585, and a 0.585≥log**_2_**-ratio≥-0.585 is considered as no change. 10: IR77298-14-1-2-B-10, 13: IR77298-14-1-2-B-13, 11: IR77298-5-6-B-11, and 18: IR77298-5-6-B-18. Click here for file

Additional file 13 **Root-specific DEGs as compared with leaf tissue of rice genotypes under two drought stress treatments**. In this table, 0.2 FTSW indicates 20 percent of fraction of transpirable soil water which is considered as severe drought stress treatment, and 0.5 FTSW indicates 50 percent of fraction of transpirable soil water which is considered as mild drought stress treatment. A transcript is considered as up-regulated (1), if log2-ratio >0.585 and down-regulated (-1) if log2-ratio <-0.585, and a 0.585≥log2-ratio≥-0.585 is considered as no change. 10: IR77298-14-1-2-B-10, 13: IR77298-14-1-2-B-13, 11: IR77298-5-6-B-11, and 18: IR77298-5-6-B-18. R: root tissue, and L: leaf tissue. Click here for file
